# Changes in dual-task cognitive performance elicited by physical exertion vary with motor task

**DOI:** 10.3389/fspor.2022.989799

**Published:** 2022-10-28

**Authors:** Jennifer A. Hogg, Christopher D. Riehm, Gary B. Wilkerson, Frank Tudini, Karissa L. Peyer, Shellie N. Acocello, Lynette M. Carlson, Tan Le, Ross Sessions, Jed A. Diekfuss, Gregory D. Myer

**Affiliations:** ^1^Department of Health and Human Performance, The University of Tennessee at Chattanooga, Chattanooga, TN, United States; ^2^Emory Sports Performance And Research Center, Flowery Branch, GA, United States; ^3^Emory Sports Medicine Center, Atlanta, GA, United States; ^4^Department of Orthopaedics, Emory University School of Medicine, Atlanta, GA, United States; ^5^Department of Physical Therapy, The University of Tennessee at Chattanooga, Chattanooga, TN, United States; ^6^Upstream Rehabilitation, Raymond, MS, United States; ^7^Cornerstone Rehabilitation, Southaven, MS, United States; ^8^The Micheli Center for Sports Injury Prevention, Waltham, MA, United States

**Keywords:** dual-task, physical activity, cognitive performance, motor performance, gait

## Abstract

**Background:**

Integrated movement and cognitive load paradigms are used to expose impairments associated with concussion and musculoskeletal injury. There is currently little information on the discriminatory nature of dual-task complexity and the relative influence of physical exertion on cognitive outcomes.

**Purpose:**

Assess cognitive performance while under motor conditions of increasing complexity before and after a standardized exercise protocol.

**Methods:**

34 participants were recruited (17 male and 17 female; 24 ± 1.4 yrs). A modified Eriksen flanker test was used to assess cognitive performance under four conditions (seated, single-leg stance, walking, and lateral stepping) before and after a 20-min moderate-to vigorous intensity treadmill protocol. The flanker test consisted of 20 sets of 5-arrow configurations, appearing in random order. To complete the response to cognitive stimulus, participants held a smartphone horizontally and were instructed to respond as quickly and as accurately as possible by tilting the device in the direction corresponding to the orientation of the middle arrow. The metrics used for analysis included average reaction time (ms), inverse efficiency index (average reaction time penalized for incorrect responses), and conflict effect (the average time cost of responding to an incongruent repetition vs. a congruent repetition). Mixed effects (condition by time) RMANOVAs were conducted to examine the effects of motor task complexity and physical exertion on cognitive performance.

**Results:**

There was a condition by time interaction for inverse efficiency index (*p* < 0.001), in which participants displayed higher cognitive efficiency for the pre-activity lateral stepping condition compared to the other three conditions (Cohen's *d* = 1.3–1.6). For reaction time and conflict effect, there were main effects for condition (*p* = 0.004 and 0.006, respectively), in which performance during lateral stepping was improved in relation to the seated condition (reaction time Cohen's *d* = 0.68; conflict effect Cohen's *d* = 0.64).

**Conclusion:**

Participants tended to display better dual-task cognitive performance under more stimulating or complex motor tasks before physical exertion, likely associated with the inverted-U arousal-performance relationship. When using dual-task assessments, clinicians should be mindful of the accompanying motor task and baseline exertion levels and their potential to disrupt or optimize cognitive performance.

## Introduction

Dual-task assessments are frequently used to identify deficits in cognitive performance associated with concussion and musculoskeletal injury risk (1–3). Dual-task paradigms involve two behaviors performed concurrently, most often a cognitive task paired with a motor task. Dual-task assessments are supported by the capacity model of attention (4), which posits that a person has a finite pool of attentional resources to manage cognitive demands. When the cognitive demand of one activity rises sufficiently it creates a bottleneck, causing the performance of the secondary task to suffer. Cognitive-motor tasks are highly relevant to sport activity. Athletes must simultaneously process stimuli, make decisions, and quickly and efficiently execute movements. For example, a basketball point guard often must concurrently predict a defender's next movement and handle the basketball while attempting to integrate into an offensive scoring strategy. Despite the applicability of cognitive-motor dual tasks to critical sport scenarios, much of the existing research in sport has been largely conducted under single-task conditions (e.g., a drop vertical jump or sidestep maneuver), though some recent work has transitioned toward the inclusion of dual-task scenarios (5).

Existing dual-task literature robustly examines the influence of cognitive load on motor task performance under a variety of conditions. This seems to be justified as deficits in motor outcomes may be more sensitive to identifying those who are at high risk of injury. However, the effects of various motor tasks on cognitive performance has gone largely underappreciated, despite potentially unique interactions that may better characterize complex sport scenarios (6). For example, a systematic review and meta-analysis (7) captured the detrimental effect of a dual task on gait in individuals who sustained sports related concussions. While this provides valuable information for understanding possible cognitive-motor impairments that are exposed by cognitive load, it does not elucidate the reciprocal effect, namely that of motor output on cognition. We believe that it is vital to understand the bidirectional relationship between motor output and cognition. Dynamic sporting activities involves a complex interplay of perceptual, cognitive and motor systems (8); however, these systems are often studied in isolation or in a unidirectional fashion (e.g., cognitive influence on motor behavior).

Understanding the conditions under which participants prioritize the cognitive or the motor component of a dual-task is critical for incorporation of dual-tasks into testing and training interventions that are appropriately representative of real on-field scenarios. On the one hand, a participant performing a relatively simple or familiar motor task may possess ample capacity to expertly complete a difficult cognitive task, but as the motor task becomes more challenging, cognitive performance may decline as the participant exhausts their remaining attentional and information processing resources. The Inverted-U hypothesis states that performance is greatest when arousal is at a moderate, optimum level (9). This hypothesis suggests that cognitive performance may increase with the difficulty of the motor task, to a point, and then begin to decline as the complimentary motor task increases with complexity.

Similarly, physical exertion has been demonstrated to improve cognition both chronically and following acute bouts (10). Greater amounts of lifestyle physical activity are associated with improvements in cognition across performance categories including academic achievement, processing speed, memory, executive function, and risk of dementia (11). Acutely, research shows a small but consistent improvement in cognition following a single bout of physical activity (12). The acute effect of activity is time- and intensity-dependent with the strongest impact on cognition appearing from 11 to 20 min after cessation of moderate physical activity (12–15). The relationship between physical activity and improved cognition is theorized to occur by increased arousal level. In agreement with the inverted-U hypothesis, the strongest effect on single-task cognition results from moderate-intensity exercise, with smaller effects for light- and vigorous-intensities (13, 15).

**Figure 1:**
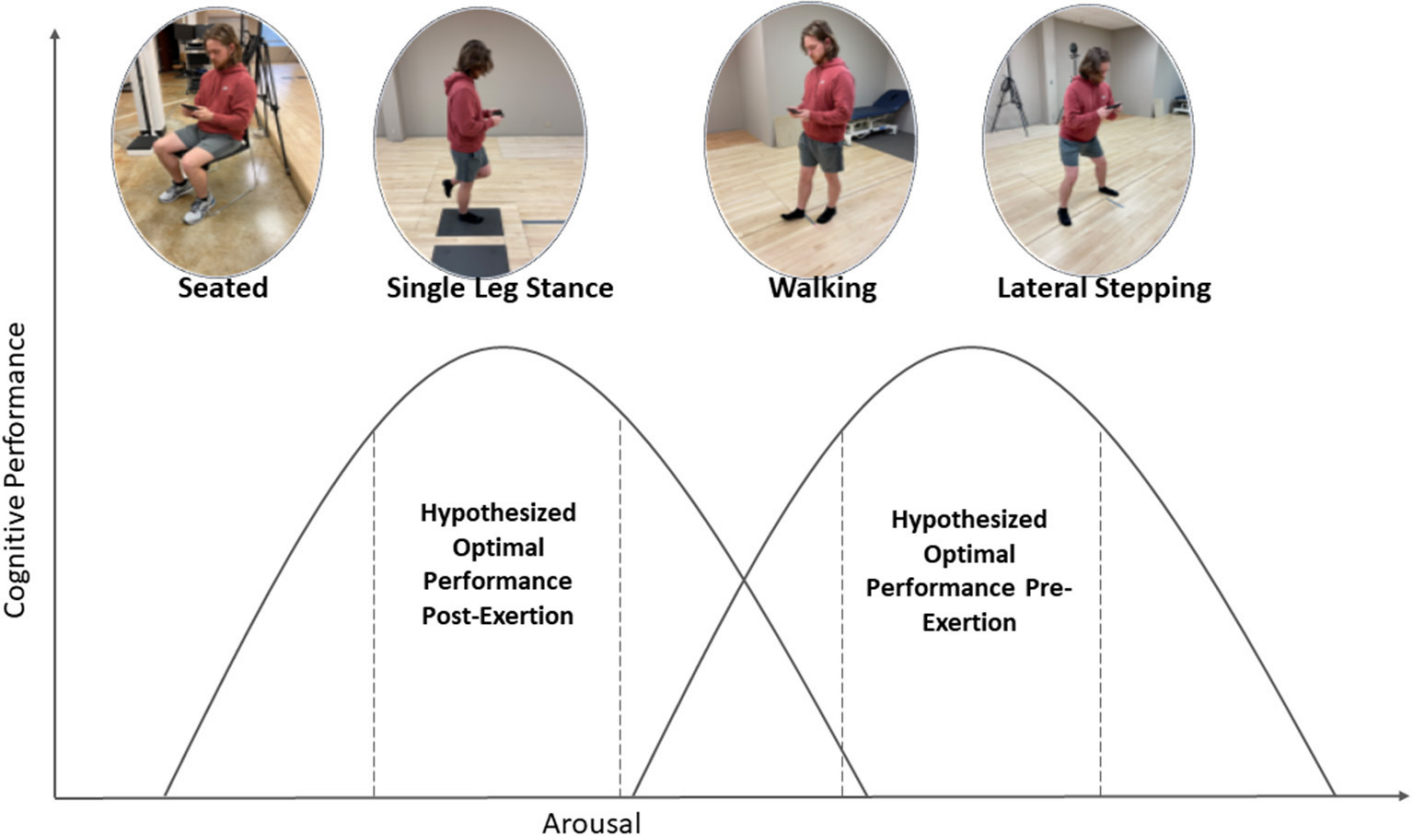
Depiction of hypothesized relationship between motor condition and cognitive performance before and after treadmill protocol. We hypothesize that physical exertion will shift the arousal-performance curve to the left relative to dual-tasking complexity.

There is currently sparse literature on the most appropriate design of dual-task assessments that assess the influence of motor task on cognitive performance, especially those that aim to manipulate arousal. Therefore, the purpose of this study was to assess cognitive performance while under motor conditions of varying complexity before and after a standardized exercise protocol. Our primary hypothesis was that a moderate-to-vigorous level of physical exertion would alter the arousal-performance relationship, such that optimum cognitive performance would be observed after exercise. Secondarily, we hypothesized that the effect of exercise would differ between various levels of motor difficulty, a possible example of which is depicted in Figure 1.

## Materials and methods

Thirty-four young healthy participants (24.03 ± 1.44 yrs) were recruited from graduate athletic training, physical therapy, and occupational therapy programs (17 females: 165.3 ± 7.1 cm, 64.9 ± 12.2 kg; 17 males: 178.7 ± 5.8 cm, 79.7 ± 12.1 kg) for a single session cross-sectional study. To maintain a sample with homogenous motor task ability, participants were excluded if they reported any persisting symptoms from any lower extremity musculoskeletal injury, any prior history of concussion, or age >30 years. All participants provided written informed consent approved by the university's Institutional Review Board.

**Figure 2:**
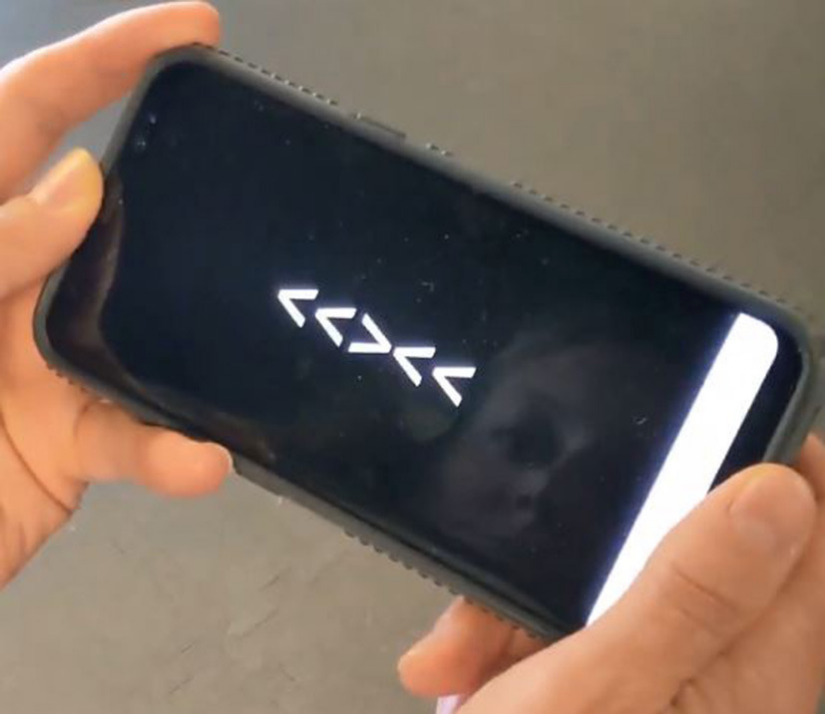
Depiction of smartphone flanker test.

The flanker task was chosen because simple manual tests of reaction time and response latency to visual stimuli do not appear to engage high-level “executive control” processes involving working memory, selective attention, decision-making, and response inhibition (16). The “flanker test” is used to quantify the slowing effect of visual distractors on response selection, which has been related to neural correlates of impaired executive function through functional magnetic resonance imaging (17–20), diffusion tensor imaging (21, 22), and electroencephalography (23–28). Previous research has demonstrated good test-retest absolute agreement on different days for both reaction time (ICC = 0.80) and response accuracy (ICC = 0.70) when administered on the smartphone (29). The flanker task (30) (Figure 2) was measured under four conditions both before and after a treadmill exercise protocol. Flanker testing was conducted *via* a smartphone application, details of which have been reported elsewhere (29). Participants were presented sequentially with a total of 20 5-arrow displays, each consisting of five arrows arranged horizontally across the smartphone screen. There were four possible arrow configurations: two congruent (< < < < < or > > > > >) and two incongruent (< < > < < or > > < > >). Each set contained 10 congruent and 10 incongruent arrow sets, randomly ordered in all conditions. Each display appeared for 300 milliseconds and displays were separated by an inter-stimulus interval randomly ranging from 500 to 1,500 milliseconds. Participants were instructed to keep their elbows at their sides and attend to the middle arrow in each display and tilt the phone as rapidly as possible in the direction corresponding to the middle arrow, a motion requiring ~20° of elbow flexion or extension. Participants were given a 10-repetition familiarization trial to ensure proper performance of the task. The flanker task was performed under four conditions in sequential order: seated with elbows at the side, single-leg standing on the self-selected dominant limb, walking at a self-selected pace over ground, and lateral stepping to the right at a self-selected pace over ground. The same order was performed for each participant.

Following baseline dual-task assessment, participants completed a treadmill protocol that was designed to impose a moderate-to-vigorous intensity aerobic demand, such as would be experienced in a practice or game and was not intended to induce a fatigued condition. The 20-min treadmill protocol consisted of a 5 min warmup, followed by a self-selected pace such that each participant attained a rating of perceived exertion (RPE) (31) of 15–18 for the final 12 min of the 20-min bout, as determined by self-report at each minute of the protocol (32). Following the exertional treadmill protocol, participants rested for ~10 min prior to completion of post-exercise dual-task assessments.

### Statistical approach

We derived three outcome measures from each flanker test. *Reaction time*, recorded in milliseconds, represented the average time from the appearance of a set of arrows until the participant tilted the smartphone at a velocity of at least 115 degrees per second. *Inverse efficiency index* combines the reaction time measure with a penalty for incorrect responses and represents the speed-accuracy tradeoff of the flanker task [reaction time + (1- accuracy proportion) × reaction time]. *Conflict effect* represents the average difference between reaction time for incongruent responses and congruent responses (incongruent average reaction time – congruent average reaction time). For all outcome measures, higher scores indicated poorer performance.

Data normality was assessed through visual inspection of histograms. Sphericity was assessed and corrected as needed with Greenhouse-Geisser corrections. Three 4x2 (condition by time) repeated-measures ANOVAs were conducted, one for each outcome measure. Tukey's *post-hoc* tests were used as appropriate. Effect sizes were computed with $\eta_{\text{p}}^{2}$ and Cohen's *d* to support interpretation. *A priori* alpha was set at *p* < 0.05. All statistical analyses were conducted in JASP (33).

## Results

Descriptive statistics for each flanker test variable are detailed in Tables 1–3. Data were not normally distributed and Greenhouse Geisser adjustments were used for all F testing. Gait speed during the walking and lateral stepping tasks were similar before and after exertional treadmill protocol (*p* = 0.80 and 0.23, respectively).

**Table 1:** Means and standard deviations for *reaction time* flanker variable for all motor conditions before and after exercise.

	**Pre-activity**	**Post-activity**
Seated	514.3 ± 61.4 ms	498.8 ± 44.8 ms
Single-leg stance	507.5 ± 46.9 ms	496.6 ± 44.7 ms
Walking	508.9 ± 55.4 ms	499.6 ± 55.0 ms
Lateral stepping	483.6 ± 61.0 ms	488.1 ± 55.2 ms

**Table 2:** Means and standard deviations for *inverse efficiency index* flanker variable for all motor conditions before and after exercise.

Seated	576.3 ± 70.4	554.7 ± 66.0
Single-leg stance	561.5 ± 56.7	547.9 ± 61.9
Walking	580.0 ± 53.7	548.5 ± 56.1
Lateral stepping	483.8 ± 61.0	564.3 ± 54.7

**Table 3:** Means and standard deviations for *conflict effect* flanker variable for all motor conditions before and after exercise.

Seated	73.7 ± 58.4 ms	53.7 ± 30.0 ms
Single-leg stance	66.8 ± 64.3 ms	41.6 ± 41.7 ms
Walking	44.2 ± 47.0 ms	44.1 ± 41.0 ms
Lateral stepping	32.4 ± 43.4 ms	37.3 ± 70.5 ms

**Figure 3:**
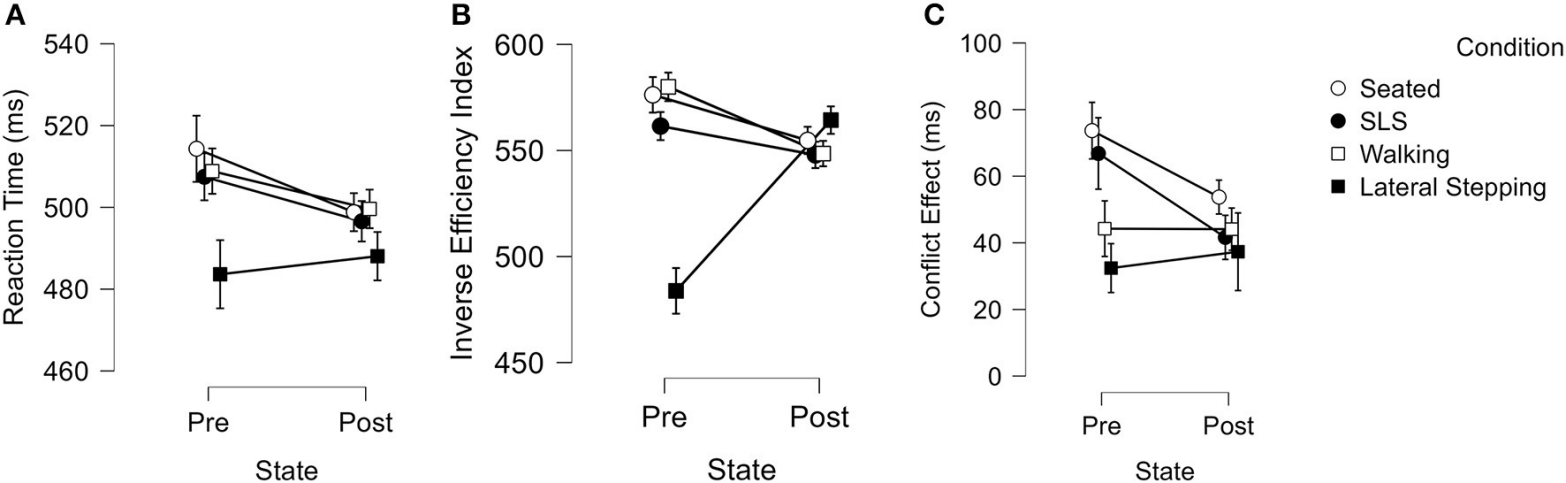
**(A–C)** Graphical representation of motor condition by pre-post activity repeated measures ANOVA results. Bars represent standard error.

For reaction time, we observed a condition main effect (Greenhouse-Geisser corrected; F_2.011_ = 6.1, *p* = 0.004; $\eta_{\text{p}}^{2}$ = 0.16), wherein lateral stepping was 16.2–20.7 ms faster than during the other three conditions (Cohen's *d* range = 0.30–0.39) (Figure 3A).

For inverse efficiency index, we observed a condition by time interaction (Greenhouse-Geisser corrected; F_2.617_ = 27.5, *p* < 0.001; $\eta_{\text{p}}^{2}$ = 0.45) and a condition main effect (Greenhouse-Geisser corrected; F_2.2_ = 15.0, *p* < 0.001; $\eta_{\text{p}}^{2}$ = 0.31). During pre-activity lateral stepping, participants displayed a lower inverse efficiency index value compared to the other motor three conditions (483.8 ± 61.0 v. 561.5 ± 56.7 – 580.0 ± 53.7; Cohen's *d* range = 1.3–1.6) (Figure 3B).

For conflict effect, we observed a condition main effect (Greenhouse Geisser corrected; F_2.6_ = 4.7, *p* = 0.006; $\eta_{\text{p}}^{2}$ = 0.13), wherein participants displayed 28.8 ms lesser conflict effect during lateral stepping compared to the seated condition (Cohen's *d* = 0.56) (Figure 3C).

## Discussion

Our primary hypothesis that optimum cognitive performance would be achieved after exercise was not supported, though we did observe trends which warrant further investigation into this theory. In general, our treadmill protocol negated pre-activity differences in cognitive performance between conditions (Figures 3A–C). Our second hypothesis, that cognitive performance would differ between various levels of motor difficulty, was supported. Specifically, the lateral stepping task displayed divergent cognitive performance. During pre-activity testing in particular, participants consistently displayed superior reaction time metrics when stepping to their right at a self-selected pace; an effect negated by the treadmill protocol.

Assessment of executive function is typically done in a rested state, but performance metrics acquired at rest only weakly associate with those acquired during moderate-intensity exercise (34). Thus, we aimed to assess the influence of a 20-min treadmill protocol on discriminatory reaction time metrics. Exercise mode, duration, and intensity are all important factors in the impact of exercise on cognition. Although both cycling and running use the aerobic energy system, cycling may produce stronger effects on cognition than does running (13), while the effects of resistance- and circuit-training on cognition are largely unexplored. The largest benefits to cognition appear in fit individuals who perform 20 min or more of physical activity (12). This suggests that the 20-min physical activity bout used in the current study was likely sufficient to induce cognitive improvements but that longer activity bouts should also be examined in the future. However, our divergent results may be explained by performing the post-exertion cognitive tasks with concurrent motor demands, as opposed to the previously reported single-task paradigm, that may have oversaturated the system/negated the robust effects of ~20 min moderate intensity exercise on cognition. Though we did observe slight, non-significant improvements in seated, single-leg stance, and walking dual-tasking from pre- to post-activity, there was appreciable worsening for the lateral stepping condition. It is possible that participants' task-related motivation levels may explain these findings by directly (or indirectly) altering adopted learning strategies (35). Task-related release of norepinephrine and dopamine is further believed to modulate brain activity in attention and information processing networks (13), which in turn is thought to drive motivation (36, 37). Though, we did not assess participants' motivation, it is possible that our participants were not engaged in the treadmill protocol to levels that would increase motivation and thus lead to detectable improvements across conditions (i.e., was not perceived as challenging enough). Alternatively, it is possible that our treadmill protocol was too fatiguing, or overstimulating, and thus hindered participant motivation to maintain cognitive performance during lateral stepping. In the current study, participants attained an RPE of 15.7 by minute 9 of the treadmill protocol, increasing to 16.5 by minute 20. An RPE of 17 is classified as “very hard” (31), and thus participants may have been too fatigued to maintain cognitive performance during the lateral stepping task.

The amount of time that elapses between the cessation of physical activity and assessment of cognitive tasks may also influence findings. A meta-analysis by Chang et al. (12) found that as exercise intensity increases, so does its effect on cognitive performance. Additionally, while performance was improved immediately after exercise for very light, light, and moderate intensity exercise, the impact on performance was greater after a delay of at least 11 min. In general, effects of physical activity began to subside following a delay of more than 20 min. These findings were corroborated by Yuxin et al. (38) who found that executive function was greatest after 10 min of rest, compared to pretest and immediately after exercise for high-intensity cycling and running as well as for moderate-intensity cycling. However, others have found larger benefits of exercise on reaction time following rest periods approaching 1 h, compared to rest of only 10 min (39). Tests of cognitive performance in the current study were evaluated 10- to 15-min following the cessation of physical activity. While this is a common measurement window for studies of exercise and impacts on cognitive performance (40, 41), further research is needed to clarify the timeline of changes in cognition following an acute bout of exercise.

We chose to study the motor tasks of sitting, single-leg stance, walking, and lateral stepping because it was thought these tasks would result in differing arousal levels, and thus result in varying cognitive performance. The inverted-U relationship between arousal and performance is a well-studied phenomenon that is thought to regulate cortical neural output per unit of sensory input (42, 43). Nevertheless, we did not observe differences between the seated, single-leg stance, or walking conditions, as demonstrated by repeated measures. It is possible these tasks were too familiar to the participants and did not cause enough arousal for improved performance. Interestingly, the lateral stepping task did elicit superior reaction time metrics for the pre-activity trial. The participants were required to tilt the phone quickly toward either the right or the left while also stepping in a rightward direction, though steps and phone tilts did not necessarily occur in-phase to one another. It is possible that the similarity in required motor behavior between the lateral stepping task and the flanker task did not impose the same bottleneck that may have suppressed cognitive performance on the other tasks. One potential neuromuscular explanation for this is that a common synergy (i.e., a single sensorimotor subsystem) (44) was required to complete both the flanker and lateral stepping task while the other task combinations required the recruitment of more heterogeneous neuromuscular components. There is some evidence that fatigue can alter synergy structure (45), so it is possible that whatever shared neuromuscular basis existed for the lateral stepping and flanker tasks was perturbed by exertion on the treadmill, resulting in a reduction of the difference in task performance. Furthermore, reaction time may be separated into pre-motor time (PMT) and motor time (MT) to separate stimulus identification and response selection (PMT) from the time to execute a chosen response (MT). A review examining these factors separately found that PMT measured during the Flanker test decreased after an acute bout of physical activity, but MT did not change significantly (46). Thus, it is reasonable that worsening of lateral stepping cognitive performance was due to alterations in PMT brought on by the exertional protocol.

Emerging evidence suggests that perceptual-cognitive capabilities are important for optimal motor control, supporting dual-task assessments that simultaneously administer perceptual-cognitive and motor tasks (47). The Eriksen flanker test is a measure of discriminatory choice reaction time and is widely accepted as a valid behavioral representation of “executive function” (17, 19, 48, 49), which collectively refers to interrelated neural processes associated with generation of goal-directed responses to sensory inputs (50). This is contrary to measures of responses to simple visual stimuli (i.e., simple reaction time), which have not been associated with activity-related change (51, 52). An additional advantage of a discriminatory cognitive task such as the flanker test is that it is possible to obtain multiple metrics from a single test. For example, slowed reaction time has consistently been identified as an important index of functional impairment in the transmission of neural signals (53), while the inverse efficiency index is a measure of the speed-accuracy tradeoff (54–56). Furthermore, the flanker task is more relevant to functional activities than more traditional language-oriented cognitive tasks that use the left hemisphere (e.g., Stroop word-color task, word memory, verbal responses to simple math problems, and backward recitation of months of the year). The right hemisphere is specialized for resolution of conflicting information and control of visuospatial attention in both the left and right visual fields, whereas the left hemisphere is specialized for language-related functions and monitoring of the body's internal state (57). The Eriksen flanker test has been shown to activate areas of the right hemisphere that are involved in detection and resolution of visual conflict (17, 58), which likely provides a cognitive challenge that is more representative of decision-making in real sport.

## Limitations

As with all exploratory studies, there are some limitations to consider for the current work. First, although our cognitive task was representative, our chosen motor tasks did not span the breadth of tasks often undertaken in real sport. Future work should use more challenging functional tasks to further test the limits of attentional capacity. Second, we did not counterbalance the order of conditions before and after the physical exertion protocol. We did include a familiarization trial which has been demonstrated to negate learning effects of the flanker task (29); however, we did observe worsening after exertion in the lateral stepping task, suggesting that learning effects did not occur. We did not assess the interrelated nature of cognitive and motor processing in the brain. Future work should design studies to explore ongoing interplay between cognition and effective motor control and assess varying cognitive tasks in addition to the flanker task. Lastly, we acknowledge that motivation, or the lack thereof, could have explained why a 20-min treadmill protocol did not uniformly elicit changes in cognitive performance across all motor conditions. Future work should consider task-related motivation and arousal as separate factors in both cognitive and motor performance.

## Conclusion

Dual-task assessments pre-activity were differentiated in that lateral-stepping resulted in improved reaction time metrics. There was no difference among post-activity assessments, suggesting that a moderate-intensity treadmill protocol eliminated discriminatory power between various flanker dual-task measures. For clinicians and researchers seeking to elicit superior cognitive dual-tasking performance assessment, a rested lateral stepping task may be preferred over sitting, single-leg stance, and forward walking.

## Data availability statement

The raw data supporting the conclusions of this article will be made available by the authors, without undue reservation.

## Ethics statement

The studies involving human participants were reviewed and approved by Institutional Review Board, The University of Tennessee at Chattanooga. The patients/participants provided their written informed consent to participate in this study. Written informed consent was obtained from the individual(s) for the publication of any potentially identifiable images or data included in this article.

## Author contributions

JH contributed to study conception, acquisition of data, data analysis, and drafting of initial and final drafts. CR contributed to initial and final drafting and final approval. GW contributed to study conception, data analysis, drafting of initial manuscript, and final approval. FT contributed to conception of study design, data acquisition, and data analysis. KP and JD contributed to manuscript development and final approval. SA contributed to study conception, acquisition of data, and final approval of manuscript. LC contributed to study conception, interpretation of results, and final approval of manuscript. TL and RS contributed to acquisition of data and interpretation of results. GM contributed to interpretation of results and final manuscript approval. All authors contributed to the article and approved the submitted version.

## Conflict of interest

Author TL was employed by Upstream Rehabilitation. Author RS was employed by Cornerstone Rehabilitation. The remaining authors declare that the research was conducted in the absence of any commercial or financial relationships that could be construed as a potential conflict of interest.

## Publisher's note

All claims expressed in this article are solely those of the authors and do not necessarily represent those of their affiliated organizations, or those of the publisher, the editors and the reviewers. Any product that may be evaluated in this article, or claim that may be made by its manufacturer, is not guaranteed or endorsed by the publisher.

## References

[1] Fischer PD Hutchison KA Becker JN Monfort SM. Evaluating the spectrum of cognitive-motor relationships during dual-task jump landing. J Appl Biomech. (2021) 37:388–95. 10.1123/jab.2020-0388 34271547

[2] Pavão SL Lima CRG Rocha NACF. Effects of motor and cognitive manipulation on the dual-task costs of center of pressure displacement in children, adolescents and young adults: a cross-sectional study. Clin Biomech. (2021) 84:1–6. 10.1016/j.clinbiomech.2021.105344 33798840

[3] Smeeton NJ Wrightson J Varga M Cowan R Schafer L. Coordination between motor and cognitive tasks in dual task gait. Gait Post. (2021) 85:138–44. 10.1016/j.gaitpost.2021.01.012 33556782

[4] Kahneman D. Attention and Effort. Vol. 1063. Englewood Cliffs, NJ: Citeseer (1973).

[5] Burcal CJ Needle AR Custer L Rosen AB. The effects of cognitive loading on motor behavior in injured individuals: a systematic review. Sports Med. (2019) 49:1233–53. 10.1007/s40279-019-01116-7 31066022

[6] Lewthwaite R Wulf G. Grand challenge for movement science and sport psychology: embracing the social-cognitive–affective–motor nature of motor behavior. Front Psychol. (2010) 1:42. 10.3389/fpsyg.2010.00042 21833211 PMC3153760

[7] Büttner F Howell DR Ardern CL Doherty C Blake C Ryan J. Concussed athletes walk slower than non-concussed athletes during cognitive-motor dual-task assessments but not during single-task assessments 2 months after sports concussion: a systematic review and meta-analysis using individual participant data. Br J Sports Med. (2020) 54:94–101. 10.1136/bjsports-2018-100164 31331944

[8] Mann DT Williams AM Ward P Janelle CM. Perceptual-cognitive expertise in sport: A meta-analysis. J Sport Exerc Psychol. (2007) 29:457–78. 10.1123/jsep.29.4.457 17968048

[9] Yerkes RM Dodson JD. The relation of strength of stimulus to rapidity of habit-formation. J Compar Neurol Psychol. (1908) 18:459–82. 10.1002/cne.920180503

[10] U.S. Department of Health and Human Services. 2018 Physical Activity Guidelines Advisory Committee Scientific Report (2018) 1–779.

[11] Stamatakis E Straker L Hamer M Gebel K. The 2018 physical activity guidelines for americans: what's new? Implications for clinicians and the public. J Orthopaed Sports Phys Therapy. (2019) 49:487–90. 10.2519/jospt.2019.0609 31258047

[12] Chang Y-K Labban JD Gapin JI Etnier JL. The effects of acute exercise on cognitive performance: a meta-analysis. Brain Res. (2012) 1453:87–101. 10.1016/j.brainres.2012.02.068 22480735

[13] Lambourne K Tomporowski P. The effect of exercise-induced arousal on cognitive task performance: a meta-regression analysis. Brain Res. (2010) 1341:12–24. 10.1016/j.brainres.2010.03.091 20381468

[14] Ludyga S Gerber M Brand S Holsboer-Trachsler E Pühse U. Acute effects of moderate aerobic exercise on specific aspects of executive function in different age and fitness groups: a meta-analysis. Psychophysiology. (2016) 53:1611–26. 10.1111/psyp.12736 27556572

[15] McMorris T Hale BJ. Differential effects of differing intensities of acute exercise on speed and accuracy of cognition: a meta-analytical investigation. Brain Cogn. (2012) 80:338–51. 10.1016/j.bandc.2012.09.001 23064033

[16] Hansen AL Johnsen BH Sollers JJ Stenvik K Thayer JF. Heart rate variability and its relation to prefrontal cognitive function: the effects of training and detraining. Eur J Appl Physiol. (2004) 93:263–72. 10.1007/s00421-004-1208-0 15338220

[17] Erickson KI Ho M-HR Colcombe SJ Kramer AF. A structural equation modeling analysis of attentional control: an event-related fMRI study. Cogn Brain Res. (2005) 22:349–57. 10.1016/j.cogbrainres.2004.09.004 15722206

[18] Fan J McCandliss BD Fossella J Flombaum JI Posner MI. The activation of attentional networks. Neuroimage. (2005) 26:471–9. 10.1016/j.neuroimage.2005.02.004 15907304

[19] Mennes M Kelly C Zuo X-N Di Martino A Biswal BB Castellanos FX. Inter-individual differences in resting-state functional connectivity predict task-induced BOLD activity. Neuroimage. (2010) 50:1690–701. 10.1016/j.neuroimage.2010.01.002 20079856 PMC2839004

[20] Zhu DC Zacks RT Slade JM. Brain activation during interference resolution in young and older adults: an fMRI study. Neuroimage. (2010) 50:810–7. 10.1016/j.neuroimage.2009.12.087 20045067 PMC2823923

[21] FitzGerald DB Crosson BA. Diffusion weighted imaging and neuropsychological correlates in adults with mild traumatic brain injury. Int J Psychophysiol. (2011) 82:79–85. 10.1016/j.ijpsycho.2011.02.011 21338633

[22] Niogi S Mukherjee P Ghajar J Johnson C Kolster R Sarkar R. Extent of microstructural white matter injury in postconcussive syndrome correlates with impaired cognitive reaction time: a 3T diffusion tensor imaging study of mild traumatic brain injury. Am J Neuroradiol. (2008) 29:967–73. 10.3174/ajnr.A0970 18272556 PMC8128563

[23] De Beaumont L Thoret H Mongeon D Messier J Leclerc S Tremblay S. Brain function decline in healthy retired athletes who sustained their last sports concussion in early adulthood. Brain. (2009) 132:695–708. 10.1093/brain/awn347 19176544

[24] Moore RD Hillman CH Broglio SP. The persistent influence of concussive injuries on cognitive control and neuroelectric function. J Athl Train. (2014) 49:24–35. 10.4085/1062-6050-49.1.01 24377962 PMC3917292

[25] Olson RL Brush CJ Ehmann PJ Buckman JF Alderman BL. A history of sport-related concussion is associated with sustained deficits in conflict and error monitoring. Int J Psychophysiol. (2018) 132:145–54. 10.1016/j.ijpsycho.2018.01.006 29355581 PMC6693583

[26] Parks AC Moore RD Wu C-T Broglio SP Covassin T Hillman CH. The association between a history of concussion and variability in behavioral and neuroelectric indices of cognition. Int J Psychophysiol. (2015) 98:426–34. 10.1016/j.ijpsycho.2015.08.006 26327621

[27] Pontifex MB O'Connor PM Broglio SP Hillman CH. The association between mild traumatic brain injury history and cognitive control. Neuropsychologia. (2009) 47:3210–6. 10.1016/j.neuropsychologia.2009.07.021 19664646

[28] Themanson JR Rosen PJ. Examining the relationships between self-efficacy, task-relevant attentional control, and task performance: evidence from event-related brain potentials. Br J Psychol. (2015) 106:253–71. 10.1111/bjop.12091 25220736

[29] Wilkerson GB Acocello SN Davis MB Ramos JM Rucker AJ Hogg JA. Wellness survey responses and smartphone app response efficiency: associations with remote history of sport-related concussion. Percept Motor Skills. (2020) 128:714–730. 10.1177/0031512520983680 33357092

[30] Eriksen BA Eriksen CW. Effects of noise letters upon the identification of a target letter in a nonsearch task. Percept Psychophys. (1974) 16:143–9. 10.3758/BF03203267

[31] Borg G. Borg's Perceived Exertion and Pain Scales. Champaign, IL: Human Kinetics. (1998).

[32] Wilkerson GB Bruce JR Wilson AW Huang N Sartipi M Acocello SN. Perceptual-motor efficiency and concussion history are prospectively associated with injury occurrences among high school and collegiate american football players. Orthopaed J Sports Med. (2021) 9:1–8. 10.1177/23259671211051722 34722788 PMC8552393

[33] JASP Team. JASP (Version 0.16.3). (2022). Available online at: https://jasp-stats.org/

[34] Faria LO Cunha FA Fortes LD Bertollo M Wanner SP Albuquerque MR. Does executive functions' performance at rest predict executive function performance during acute physical exercise? Int J Sport Exerc Psychol. (2021) 20:1490–1506. 10.1080/1612197X.2021.1956569

[35] Kyndt E Dochy F Struyven K Cascallar E. The direct and indirect effect of motivation for learning on students' approaches to learning through the perceptions of workload and task complexity. High Educ Res Dev. (2011) 30:135–50. 10.1080/07294360.2010.501329

[36] Koepp MJ Gunn RN Lawrence AD Cunningham VJ Dagher A Jones T. Evidence for striatal dopamine release during a video game. Nature. (1998) 393:266–8. 10.1038/30498 9607763

[37] Schultz W. Updating dopamine reward signals. Curr Opin Neurobiol. (2013) 23:229–38. 10.1016/j.conb.2012.11.012 23267662 PMC3866681

[38] Yuxin Z Fenghua S Chiu MM Siu AY-S. Effects of high-intensity interval exercise and moderate-intensity continuous exercise on executive function of healthy young males. Physiol Behav. (2021) 239:113505. 10.1016/j.physbeh.2021.113505 34153324

[39] Rattray B Smee D. Exercise improves reaction time without compromising accuracy in a novel easy-to-administer tablet-based cognitive task. J Sci Med Sport. (2013) 16:567–70. 10.1016/j.jsams.2012.12.007 23337198

[40] Chang YK Chu CH Wang CC Song TF Wei GX. Effect of acute exercise and cardiovascular fitness on cognitive function: an event-related cortical desynchronization study. Psychophysiology. (2015) 52:342–51. 10.1111/psyp.12364 25308605

[41] Voss MW Weng TB Narayana-Kumanan K Cole RC Wharff C Reist L. Acute exercise effects predict training change in cognition and connectivity. Med Sci Sports Exerc. (2020) 52:131. 10.1249/MSS.0000000000002115 31385912 PMC7753185

[42] Corbetta M Patel G Shulman GL. The reorienting system of the human brain: from environment to theory of mind. Neuron. (2008) 58:306–24. 10.1016/j.neuron.2008.04.017 18466742 PMC2441869

[43] Waschke L Kloosterman NA Obleser J Garrett DD. Behavior needs neural variability. Neuron. (2021) 109:751–66. 10.1016/j.neuron.2021.01.023 33596406

[44] Kuznetsov NA Shockley KD Richardson MJ Riley MA. Effect of precision aiming on respiration and the postural-respiratory synergy. Neurosci Lett. (2011) 502:13–7. 10.1016/j.neulet.2011.07.012 21798313

[45] Hajiloo B Anbarian M Esmaeili H Mirzapour M. The effects of fatigue on synergy of selected lower limb muscles during running. J Biomech. (2020) 103:109692. 10.1016/j.jbiomech.2020.109692 32151383

[46] J Alibazi R Kidgell D Zoghi M Jaberzadeh S. What are the acute effects of aerobic exercise on fractionated response time: a systematic review and meta-analysis. J Sci Sport Exerc. (2020) 2:97–112. 10.1007/s42978-019-0026-3

[47] Chmielewski TL Tatman J Suzuki S Horodyski M Reisman DS Bauer RM. Impaired motor control after sport-related concussion could increase risk for musculoskeletal injury: implications for clinical management and rehabilitation. J Sport Health Sci. (2021) 10:154–61. 10.1016/j.jshs.2020.11.005 33188963 PMC7987572

[48] Kelly AC Uddin LQ Biswal BB Castellanos FX Milham MP. Competition between functional brain networks mediates behavioral variability. Neuroimage. (2008) 39:527–37. 10.1016/j.neuroimage.2007.08.008 17919929

[49] St George RJ Jayakody O Healey R Breslin M Hinder MR Callisaya ML. Cognitive inhibition tasks interfere with dual-task walking and increase prefrontal cortical activity more than working memory tasks in young and older adults. Gait Post. (2022) 95:186–91. 10.1016/j.gaitpost.2022.04.021 35525151

[50] Diamond A. Executive functions. Annu Rev Psychol. (2013) 64:135. 10.1146/annurev-psych-113011-143750 23020641 PMC4084861

[51] Heath M Petrella A Blazevic J Lim D Pelletier A Belfry GR. A post-exercise facilitation of executive function is independent of aerobically supported metabolic costs. Neuropsychologia. (2018) 120:65–74. 10.1016/j.neuropsychologia,0.2018.10.002 30321613

[52] Kamijo K Nishihira Y Higashiura T Kuroiwa K. The interactive effect of exercise intensity and task difficulty on human cognitive processing. Int J Psychophysiol. (2007) 65:114–21. 10.1016/j.ijpsycho.2007.04.001 17482699

[53] Filley CM Fields RD. White matter and cognition: making the connection. J Neurophysiol. (2016) 116:2093–104. 10.1152/jn.00221.2016 27512019 PMC5102321

[54] Bruyer R Brysbaert M. Combining speed and accuracy in cognitive psychology: is the inverse efficiency score (IES) a better dependent variable than the mean reaction time (RT) and the percentage of errors (PE)? Psychol Belg. (2011) 51:5–13. 10.5334/pb-51-1-5

[55] Rach S Diederich A Colonius H. On quantifying multisensory interaction effects in reaction time and detection rate. Psychol Res. (2011) 75:77–94. 10.1007/s00426-010-0289-0 20512352

[56] Vandierendonck A. A comparison of methods to combine speed and accuracy measures of performance: a rejoinder on the binning procedure. Behav Res Methods. (2017) 49:653–73. 10.3758/s13428-016-0721-5 26944576

[57] Serrien DJ Ivry RB Swinnen SP. Dynamics of hemispheric specialization and integration in the context of motor control. Nat Rev Neurosci. (2006) 7:160–6. 10.1038/nrn1849 16429125

[58] Nee DE Wager TD Jonides J. Interference resolution: insights from a meta-analysis of neuroimaging tasks. Cogn Affect Behav Neurosci. (2007) 7:1–17. 10.3758/CABN.7.1.1 17598730

